# Being Left Alone at Home and Dental Caries of Children Aged 6–7 Years

**DOI:** 10.2188/jea.JE20210321

**Published:** 2023-06-05

**Authors:** Yusuke Matsuyama, Aya Isumi, Satomi Doi, Takeo Fujiwara

**Affiliations:** 1 Department of Global Health Promotion, Tokyo Medical and Dental University, Tokyo, Japan; 2 Japan Society for Promotion of Science, Tokyo, Japan

**Keywords:** child dentistry, epidemiology, dental public health, dental caries

## Abstract

**Background:**

Leaving children alone at home is considered child neglect in some countries but is not prohibited in Japan. We investigated the association between being left alone at home and dental caries of children aged 6–7 years in Japan.

**Methods:**

The data on first graders in all 69 public elementary schools in Adachi, Tokyo, obtained from repeated cross-sectional surveys in 2015, 2017, and 2019 were analyzed. Caregivers answered the questionnaire, and the data were linked to the information on children’s dental caries evaluated in school dental health checkups (*N* = 12,029). Poisson regression analysis with propensity score matching (PSM) was applied to account for confounders.

**Results:**

Leaving children alone at home for ≥1 hour during the weekdays was reported by 46.4% of the caregivers, which did not vary across years. The PSM analysis showed that, compared with children never being left alone at home, children being left alone at home for ≥1 time per week had more dental caries (mean ratio [MR] 1.11; 95% confidence interval (CI), 1.02–1.21; *P* = 0.016), while <1 time per week was not associated (MR 0.97; 95% CI, 0.92–1.03; *P* = 0.345). The difference between those being left alone at home for <1 time per week and those being left alone for ≥1 time per week was not significant after applying Bonferroni correction (MR 1.12; 95% CI, 1.00–1.26; *P* = 0.041).

**Conclusion:**

Leaving children alone at home for ≥1 hour every week might be a risk factor for dental caries of children aged 6–7 years.

## INTRODUCTION

Dental caries is the most prevalent disease globally, affecting those from the early stage of life.^1^ Dental caries affects the daily life activities of children, such as eating, sleeping, and smiling, owing to the increase of pain, discomfort, and worsening appearance.^2^ Family environments, including parental practices, are important determinants of the dental caries of children^3^^,^^4^; for example, the lack of supervision of caregivers on the health behaviors of children is associated with dental caries after controlling for family socioeconomic status.^5^

Leaving children alone at home has been identified as one of the most common types of child maltreatment,^6^^,^^7^ in spite of legal repercussions in some countries.^8^^–^^10^ For instance, a national survey in the United States reported that 15% of children aged 6–12 spend time alone or with their young siblings.^11^ It is often demanding for parents to keep their children under safety and supervision while coordinating their daily schedules. Parents and caregivers commonly face complex decision-making whether a child is ready to be left home because age and other factors, such as the maturity of the child and sibling existence, contribute to it.

While some studies have suggested that staying alone at home could provide children opportunities to foster self-confidence and independence,^12^^,^^13^ most studies have reported the association between being left alone at home and unfavorable health and behavioral outcomes.^7^^,^^14^^–^^16^ Being left alone at home is associated with behavior problems in school children in Japan.^16^^,^^17^ Delinquency behaviors, such as smoking and alcohol drinking, are also high if children spend time without adult supervision.^14^^,^^15^ Moreover, the experience of being left at home in childhood is associated with poor mental and physical health in young adulthood.^7^

Given these backgrounds, being left alone at home may be a risk factor for dental caries. Children may show unhealthy oral health-related behaviors, such as high sugar consumption, when they are not under adult supervision. One study has verified that snack and juice consumption is high among children with lower parental monitoring.^18^ Thus, sugar intake tends to increase when children are frequently left alone at home, as they spent more time without a caregiver’s supervision. Accordingly, this could entail the continuing risk of a child’s dental caries. However, to the best of our knowledge, no study has yet explored this relationship of a child’s oral health with being left alone at home.

Child dental caries remain prevalent in Japan; in fact, 21.7% of elementary school children suffered from untreated dental caries in 2019,^19^ making it crucial to determine its risk factors in the country. There is no law in Japan that prohibits leaving children alone at home, so by implementing one, the risk for child dental caries can be potentially decreased. A study reported that 47.8% of caregivers leave their first-grade children alone at home.^17^ The present study aimed to investigate the association between being left alone at home and dental caries of first graders (ie, aged 6–7 years) in elementary schools in Japan.

## METHODS

### Study participants

We conducted a cross-sectional study by analyzing three waves of repeated cross-sectional data from the Adachi Child Health Impact of Living Difficulty (A-CHILD) study in 2015, 2017, and 2019. The cohort profile of the A-CHILD study participants is reported elsewhere.^20^ At each survey, the questionnaires were distributed to the caregivers of first graders in all 69 public elementary schools in Adachi, Tokyo, Japan. The survey data were also linked to the information on the health status of children, including dental caries, obtained from the annual school health checkup. The surveys were answered by 4,291 (valid response rate of 80.1%), 4,208 (valid response rate of 81.6%), and 4,042 (valid response rate of 78.8%) of caregivers in 2015, 2017, and 2019, respectively. After excluding children with missing information on age (*n* = 328), sex (*n* = 15), dental caries status (*n* = 107), or frequency of being left alone at home (*n* = 78), the data of 12,029 children (9,152 of them were complete cases) were included in the analysis. Informed consent was obtained from the caregivers in opt-out formats. The present study was approved by the Ethics Committee at Tokyo Medical and Dental University (M2016-284-02).

### Outcome variable: dental caries experience

In Japan, mandatory school health checkups, including dental health checkups by school dentists, are conducted annually. As school checkups are conducted nationwide in Japan, the dentists were not calibrated because of a feasibility issue. Instead, they were required to follow the national guideline.^21^ A dental mirror and ball-ended probe or the Community Periodontal Index probe was used under sufficient light in schools, and dental units and radiography were not employed. The present study utilized the data on the dental caries experience (ie, decayed or filled teeth) of children in primary and permanent teeth as the outcome.

### Exposure variable: being left alone at home

The caregivers answered how often they leave their children alone at home ≥1 hour during the weekdays with the options of “never,” “1 to 11 times a year,” “1 to 3 times a month,” and “once a week or more.” We followed a previous study^17^ and categorized the variable into never, <1 time per week, and ≥1 time per week.

### Covariates

Following previous studies,^5^^,^^17^ the following variables, assessed using the questionnaires, were considered as confounders: survey year, child’s age and sex, marital status of parents, mother’s age, mother’s educational attainment, mother’s employment status, annual household income, survey respondent, having older siblings, having younger siblings, living with grandparents, and caregiver’s psychological distress measured with Kessler Psychological Distress Scale (K6) scores.^22^ Table 1 presents the details of these variables.

**Table 1.** Demographic characteristics of the study participants (complete cases; *N* = 9,152)

		Being left alone at home		

	Total (*N* = 9,152)	Never (*N* = 4,903; 53.6%)	<1/w (*N* = 3,386; 37.0%)	≥1/w (*N* = 863; 9.4%)

	*N* (%) or mean (SD)	*N* (%) or mean (SD)	*N* (%) or mean (SD)	*N* (%) or mean (SD)
Dental caries experience	1.26 (2.39)	1.15 (2.26)	1.34 (2.48)	1.60 (2.66)
Survey year				
2015	2,892 (31.6%)	1,502 (30.6%)	1,114 (32.9%)	276 (32.0%)
2017	3,195 (34.9%)	1,694 (34.6%)	1,200 (35.4%)	301 (34.9%)
2019	3,065 (33.5%)	1,707 (34.8%)	1,072 (31.7%)	286 (33.1%)
Age, months	84.83 (3.47)	84.71 (3.48)	84.90 (3.45)	85.27 (3.41)
Sex				
Boy	4,686 (51.2%)	2,415 (49.3%)	1,791 (52.9%)	480 (55.6%)
Girl	4,466 (48.8%)	2,488 (50.7%)	1,595 (47.1%)	383 (44.4%)
Marital status of parents				
Married	8,492 (92.8%)	4,552 (92.8%)	3,174 (93.7%)	766 (88.8%)
Unmarried	660 (7.2%)	351 (7.2%)	212 (6.3%)	97 (11.2%)
Mother’s age, years				
<30	477 (5.2%)	250 (5.1%)	178 (5.3%)	49 (5.7%)
30–34	1,656 (18.1%)	865 (17.6%)	627 (18.5%)	164 (19.0%)
35–39	3,151 (34.4%)	1,715 (35.0%)	1,146 (33.8%)	290 (33.6%)
40–44	2,893 (31.6%)	1,523 (31.1%)	1,095 (32.3%)	275 (31.9%)
≥45	975 (10.7%)	550 (11.2%)	340 (10.0%)	85 (9.8%)
Mother’s educational attainment				
High school or less	2,865 (31.3%)	1,474 (30.1%)	1,095 (32.3%)	296 (34.3%)
Some college	3,808 (41.6%)	1,995 (40.7%)	1,443 (42.6%)	370 (42.9%)
College or more	2,410 (26.3%)	1,403 (28.6%)	820 (24.2%)	187 (21.7%)
Other/unknown	69 (0.8%)	31 (0.6%)	28 (0.8%)	10 (1.2%)
Mother’s employment status				
Full-time/part-time	5,680 (62.1%)	2,998 (61.1%)	1,984 (58.6%)	698 (80.9%)
Self-employed	458 (5.0%)	240 (4.9%)	176 (5.2%)	42 (4.9%)
Not working	3,014 (32.9%)	1,665 (34.0%)	1,226 (36.2%)	123 (14.3%)
Annual household income				
<3.0 million JPY	854 (9.3%)	445 (9.1%)	311 (9.2%)	98 (11.4%)
3.0–5.9 million JPY	3,534 (38.6%)	1,875 (38.2%)	1,325 (39.1%)	334 (38.7%)
6.0–9.9 million JPY	3,110 (34.0%)	1,650 (33.7%)	1,163 (34.3%)	297 (34.4%)
≥10.0 million JPY	1,014 (11.1%)	588 (12.0%)	338 (10.0%)	88 (10.2%)
Unknown	640 (7.0%)	345 (7.0%)	249 (7.4%)	46 (5.3%)
Survey respondent				
Mother	8,514 (93.0%)	4,511 (92.0%)	3,195 (94.4%)	808 (93.6%)
Father	619 (6.8%)	382 (7.8%)	184 (5.4%)	53 (6.1%)
Other	19 (0.2%)	10 (0.2%)	7 (0.2%)	2 (0.2%)
Having older siblings				
No	4,901 (53.6%)	3,414 (69.6%)	1,274 (37.6%)	213 (24.7%)
Yes	4,251 (46.4%)	1,489 (30.4%)	2,112 (62.4%)	650 (75.3%)
Having younger siblings				
No	4,966 (54.3%)	2,461 (50.2%)	1,954 (57.7%)	551 (63.8%)
Yes	4,186 (45.7%)	2,442 (49.8%)	1,432 (42.3%)	312 (36.2%)
Living with grandparents				
No	8,287 (90.5%)	4,321 (88.1%)	3,144 (92.9%)	822 (95.2%)
Yes	865 (9.5%)	582 (11.9%)	242 (7.1%)	41 (4.8%)
Caregiver’s K6 score	3.57 (4.33)	3.31 (4.18)	3.67 (4.23)	4.61 (5.30)
Factor: poor involvement	−0.00 (1.00)	−0.10 (0.97)	0.05 (0.99)	0.40 (1.10)
Factor: child abuse	−0.00 (1.00)	−0.08 (0.92)	0.04 (1.01)	0.30 (1.28)
Factor: lack of supervision on child’s health behaviors	0.00 (1.00)	−0.10 (0.98)	0.05 (0.99)	0.38 (1.04)
Snack eating habit				
Never	389 (4.3%)	229 (4.7%)	121 (3.6%)	39 (4.5%)
Eat on time	6,639 (72.5%)	3,590 (73.2%)	2,501 (73.9%)	548 (63.5%)
Help oneself	2,124 (23.2%)	1,084 (22.1%)	764 (22.6%)	276 (32.0%)
Frequency of drinking juice				
Never or several times per month	1,765 (19.3%)	984 (20.1%)	632 (18.7%)	149 (17.3%)
Several times per week	5,642 (61.6%)	3,011 (61.4%)	2,090 (61.7%)	541 (62.7%)
Everyday	1,745 (19.1%)	908 (18.5%)	664 (19.6%)	173 (20.0%)

JPY, Japanese yen (110 JPY = 1 United States dollar); SD, standard deviation.

^a^Chi-square test for categorical variables and *t*-test for continuous variables.

It was expected that leaving children alone at home is correlated with other parenting practices, such as neglect. To account for this, we followed a previous study^5^ and derived factor scores on three aspects of poor parenting by incorporating specific parenting practices. eTable 1 exhibits the list of the variables, and eTable 2 displays the results from the factor analysis. We first performed exploratory factor analysis to evaluate the underlying dimensionality of the parenting practices. We then confirmed that the same three dimensions (ie, poor involvement, child abuse, and lack of supervision of a child’s health behavior) as the previous study^5^ were observed. Thereafter, we performed confirmatory factor analysis to evaluate the extent to which the models fit our data. The results showed good model fit (comparative fit index, 0.932; root mean square error of approximation, 0.025; and standardized root mean square residual, 0.022). In the following analysis, the z-scores for each factor score derived from the confirmatory factor analysis were employed as confounders.

The snack eating habits and frequency of drinking juice of the children answered by the caregivers were also included in the analysis as potential mediators. The snack eating habits were assessed with the question “When does your child eat snacks?”, with the response items of “does not eat snacks,” “eats snacks at a set time,” and “eats snacks freely whenever he/she wants.” The frequency of drinking juice was assessed with the question “How often does your child drink sugar-sweetened beverages?”, with the response items of “several times per month or never,” “once per week,” “2–3 times per week,” “4–6 times per week,” “once a day,” and “twice or more per day.” The variable was grouped into “never or several times per month,” “several times per week,” and “every day.”

### Statistical analysis

Poisson regression models were fitted to evaluate the association between being left alone at home and the number of dental caries. Two models were also constructed; namely, a model adjusting for all confounders (model 1) and a model further including snack eating habits and frequency of drinking juice to evaluate whether oral health-related behaviors explain the association between being left alone at home and dental caries (model 2).

Propensity score matching (PSM) was performed to consider the confounding.^23^ Three sets of matching were conducted: (1) between children never being left alone at home and those being left <1 time per week, (2) between children never being left alone at home and those being left ≥1 time per week, and (3) between children being left alone at home <1 time per week and those being left ≥1 per week. Bonferroni correction for multiple comparisons was applied (ie, *P* < 0.017 was considered statistically significant). The propensity scores were estimated using logistic regression with all confounders. One-to-one optimal matching with no replacements with a caliper of 0.2 standard deviation of the propensity scores in the logit scale was performed (ie, 0.16 to match never and <1 time per week; 0.28 to never and ≥1 time per week; and 0.16 to match <1 time and ≥1 time per week). Missing information on covariates was imputed using multiple imputation by chained equations under missing-at-random assumptions, and 20 imputed data sets were created and combined the estimates using Rubin’s combination methods.^24^ As eTable 3 reports, the proportion of missing data ranged from 0–6.6% across the variables. As a robustness check, multivariable regression adjustments and complete case analyses were also performed. Further, an auxiliary analysis was also conducted to examine the association between being left alone at home and dental caries in permanent teeth and oral health-related behaviors. We followed Strengthening the Reporting of Observational studies in Epidemiology (STROBE) guidelines.

## RESULTS

**Figure 1.**
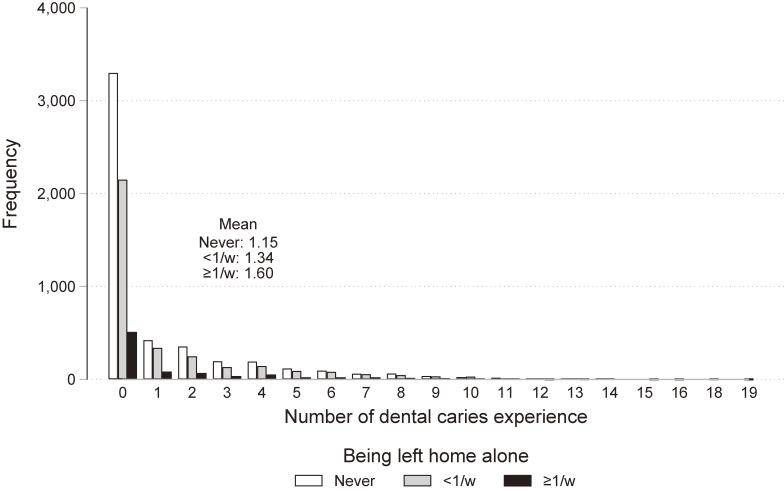
Distribution of dental caries experience by the frequency of being left alone at home (complete cases; *N* = 9,152)

Table 1 describes the demographic characteristics of the respondents without missing data. Of the caregivers, 37.0% and 9.4% reported leaving their children alone at home <1 or ≥1 time per week, respectively. As Figure 1 illustrates, the number of dental caries experience was greater among children being left alone at home. The prevalence of dental caries was 34.9%. Boys, children with older age, children with lower maternal educational attainment, children having older siblings, children not having younger siblings, children not living with grandparents, children whose caregivers had higher psychological distress, children with poor parental involvement, children who experience child abuse, and children whose health behaviors lack supervision were more likely to be left alone at home.

**Figure 2.**
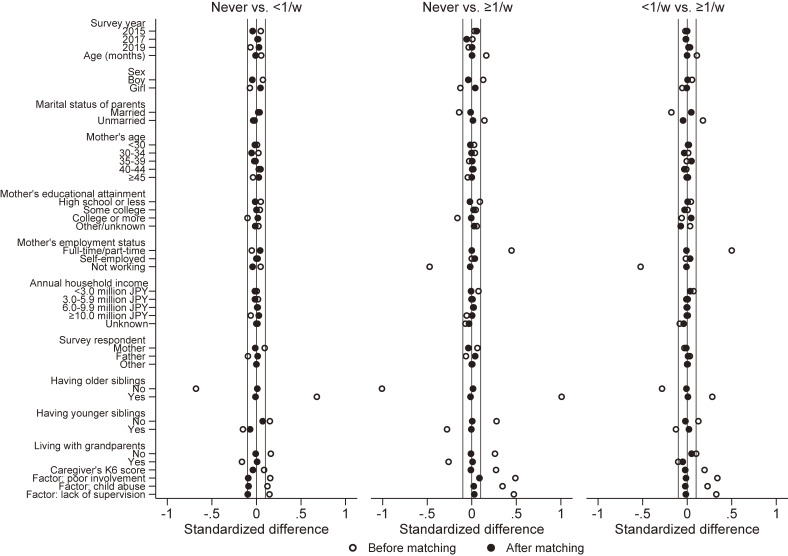
Covariate balance plot for complete cases. Vertical lines indicate −0.1, 0, and 0.1, respectively.

Table 2 compares the demographic characteristics of children following PSM, with the subsequent pairings: (1) 2,682 pairs of children who were never left alone at home and those who were left alone <1 time per week, (2) 817 pairs of children who were never left alone at home and those who were left along ≥1 time per week, and (3) 841 pairs of children who were left alone at home <1 time per week and those who were left alone ≥1 per week. As shown in Figure 2, the covariate balance improved after the matching, and the standardized differences were <0.1 for every confounder.

**Table 2.** Demographic characteristics of propensity score-matched study participants (complete cases)

	Being left alone at home					
	Never vs <1/w (*N* of pairs = 2,682)		Never vs ≥1/w (*N* of pairs = 817)		<1/w vs ≥1/w (*N* of pairs = 841)	

	Never	<1/w	Never	≥1/w	<1/w	≥1/w

	% or mean	% or mean	% or mean	% or mean	% or mean	% or mean
Survey year						
2015	32.7%	30.8%	28.9%	31.5%	32.0%	32.0%
2017	34.5%	35.0%	37.1%	34.4%	35.1%	34.4%
2019	32.8%	34.2%	34.0%	34.1%	32.9%	33.7%
Age, months	84.80	84.77	85.25	85.26	85.24	85.24
Sex						
Boy	51.6%	49.3%	56.7%	54.7%	55.1%	55.3%
Girl	48.4%	50.7%	43.3%	45.3%	44.9%	44.7%
Marital status of parents						
Married	93.1%	93.6%	90.1%	89.7%	88.5%	89.8%
Unmarried	6.9%	6.4%	9.9%	10.3%	11.5%	10.2%
Mother’s age, years						
<30	5.6%	5.2%	6.0%	5.6%	5.4%	5.6%
30–34	18.1%	16.0%	18.2%	18.1%	20.0%	18.7%
35–39	33.9%	33.4%	33.5%	33.8%	31.5%	33.8%
40–44	31.2%	33.4%	31.9%	32.2%	33.4%	32.0%
≥45	11.2%	12.0%	10.3%	10.3%	9.8%	10.0%
Mother’s educational attainment						
High school or less	32.4%	31.8%	35.1%	34.1%	33.9%	34.1%
Some college	40.9%	41.0%	41.2%	42.2%	44.2%	42.8%
College or more	25.8%	26.5%	22.9%	22.6%	20.0%	21.9%
Other/unknown	0.8%	0.7%	0.7%	1.0%	1.9%	1.2%
Mother’s employment status						
Full-time/part-time	58.5%	60.6%	79.9%	79.9%	80.7%	80.4%
Self-employed	5.1%	5.1%	4.3%	5.0%	4.3%	5.0%
Not working	36.4%	34.4%	15.8%	15.1%	15.0%	14.6%
Annual household income						
<3.0 million JPY	9.7%	9.2%	11.1%	10.9%	9.8%	10.8%
3.0–5.9 million JPY	39.0%	38.2%	38.4%	38.3%	38.6%	38.9%
6.0–9.9 million JPY	33.6%	34.0%	33.8%	34.8%	34.8%	34.6%
≥10.0 million JPY	10.4%	11.3%	10.3%	10.4%	10.3%	10.2%
Unknown	7.3%	7.3%	6.4%	5.6%	6.4%	5.5%
Survey respondent						
Mother	93.6%	93.3%	94.2%	93.3%	93.8%	93.6%
Father	6.2%	6.6%	5.5%	6.5%	5.9%	6.2%
Other	0.2%	0.2%	0.2%	0.2%	0.2%	0.2%
Having older siblings						
No	47.0%	47.5%	25.5%	26.1%	25.7%	25.3%
Yes	53.0%	52.5%	74.5%	73.9%	74.3%	74.7%
Having younger siblings						
No	54.7%	58.2%	64.3%	64.5%	64.9%	64.0%
Yes	45.3%	41.8%	35.7%	35.5%	35.1%	36.0%
Living with grandparents						
No	91.3%	91.1%	95.2%	95.0%	93.9%	95.1%
Yes	8.7%	8.9%	4.8%	5.0%	6.1%	4.9%
Caregiver’s K6 score	3.47	3.31	4.25	4.20	4.48	4.37
Factor: poor involvement, mean	0.01	−0.08	0.24	0.33	0.37	0.36
Factor: child abuse, mean	0.00	−0.09	0.17	0.19	0.28	0.25
Factor: lack of supervision on child’s health, mean	0.01	−0.09	0.27	0.30	0.35	0.33

JPY, Japanese yen (110 JPY = 1 United States dollar).

**Figure 3.**
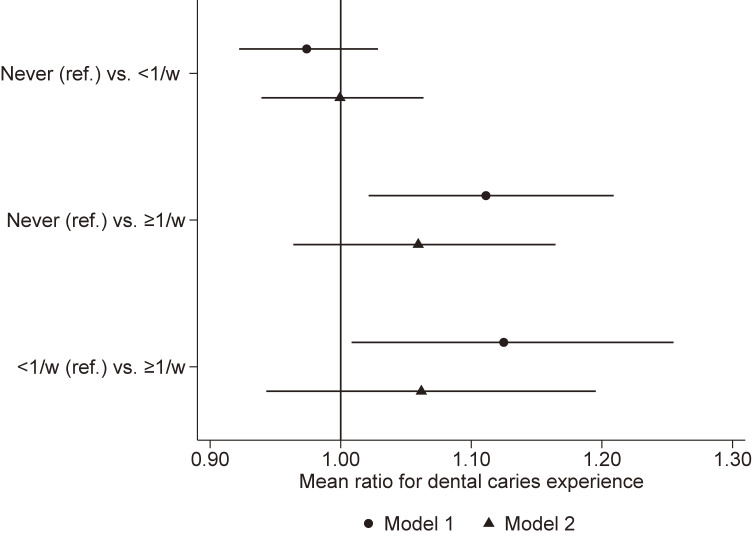
Association between being left alone at home and dental caries in children. Results from Poisson regression analysis with propensity score matching after multiple imputation; model 1 shows crude estimates, whereas model 2 shows estimates controlled for snack eating habits and frequency of drinking juice as explanatory variables

Figure 3 depicts the association between being left alone at home and dental caries of children. We applied Bonferroni correction to consider multiple comparisons (ie, *P* < 0.017 was considered statistically significant). Dental caries experience was not significantly different between children never being left alone at home and those being left <1 time per week (MR 0.97; 95% CI, 0.92–1.03; *P* = 0.345). Children being left alone at home for ≥1 time per week had more dental caries than those never being left (MR 1.11; 95% CI, 1.02–1.21; *P* = 0.016). Dental caries experience was not significantly different between children being left alone at home for <1 time per week and those being left ≥1 time per week after applying the Bonferroni correction (MR 1.12; 95% CI, 1.00–1.26; *P* = 0.041). As model 2 for each comparison shows, the observed difference was explained by snack eating habits and the frequency of drinking juice.

The results of the robustness check and auxiliary analysis are reported in supplementary materials. Similar estimates were observed in the multivariable regression adjustment (eTable 4) and PSM with complete cases (eTable 5). Children that were left alone at home were more likely to consume snacks whenever they wished, but no significant difference was observed with regard to the frequency of drinking juice (eTable 6). The association between being left alone at home and dental caries in permanent teeth was not significant (eTable 7).

## DISCUSSION

The present study is the first to find an association between being left alone at home and dental caries of children. We utilized the population-based data of 12,029 first graders from multiple years of surveys whose dental status was evaluated by school dentists. The PSM analysis suggests that children being left alone at home for ≥1 time per week had more dental caries than those never being left alone at home, whereas there was no significant difference in dental caries between children being left alone at home for <1 time per week and those never being left alone at home. The difference between those being left alone at home for <1 time per week and those being left alone at home for ≥1 time per week was not significant after applying Bonferroni correction. The oral health-related behaviors of children (ie, snack eating habits and the frequency of drinking juice) explained the association between being left alone at home and having dental caries.

The prevalence of dental caries among participants included in the present study was 34.9%, which is lower than the national average for 6-year-olds (41.5% in 2017).^25^ Similarly, in the United States, prevalence of dental caries was 50.5% in 2015 for children aged 6–12 years.^26^ The percentage of children being left alone at home varies by country. In this study, 37.0% and 9.4% of children had been left alone at home <1 time per week and ≥1 time per week, respectively. In the United States, 15% of children aged 6–12 were reported as spending time without adult supervision, even though leaving children alone at home is prohibited by the law.^11^ On the other hand, in South Korea, with the absence of such a law, 18.4% of children aged 9–12 years are reported to stay alone at home for 3 days per week or more.^27^ Adults leaving children alone at home is often influenced by the law or guidelines, parental working environments, and cultural differences related to social norms and neighborhood security. Therefore, the relationship between leaving children alone at home and their dental caries can vary by country. For example, children who were left alone at home despite lawful repercussions could have more dental caries and poor oral conditions because their caregivers’ parenting practices and living environments might be marginalized. This association can also be influenced by other determinants of dental caries, such as high availability of sugar, lack of water fluoridation, and poor access to dental healthcare.

Neglectful parenting is a risk for dental caries of children.^3^^,^^5^^,^^28^ Valencia-Rojas et al reported that the prevalence of dental caries in children under social protection in Toronto was 56%, which is higher than the general population of 5-year-olds in that city.^28^ Less severe neglect could also be a risk of child dental caries owing to a lack of proper care.^5^ However, the present study found no significant differences in dental caries between children who were never left alone at home and those that were left <1 time per week. This suggests that the caregivers generally engage in proper parenting, but sometimes have to leave their children alone at home because of urgent or unforeseen needs. Leaving children alone at home <1 time per week may not increase child dental caries, a finding which is in line with previous studies that found an association between adverse mental health outcomes in children on being left alone at home ≥1, but not <1, times per week.^17^

As we considered poor parenting practices as covariates, the association related to being left alone at home would be independent of other neglectful parenting. Oral health-related behaviors, therefore, are likely to explain the direct relationship between being left alone at home and dental caries. As shown in our auxiliary analysis, children who were left alone at home frequently had less favorable snack-consuming habits but no significant difference in terms of the frequency of drinking juice. Thus, the observed relationship between being left alone at home and dental caries would be mainly explained by snack eating habits. Young children can feel fear and have less trust in their caregivers when they are left alone at home,^13^ which may lead to a poor parent-child relationship and be a potential barrier for the child to learn healthy behaviors from caregivers. Further studies are needed to investigate how children who are left alone at home develop unfavorable oral health-related behaviors.

In Japan, no guidelines help caregivers decide whether a child is ready to stay alone at home. Nevertheless, first graders in elementary schools (ie, aged 6–7 years) do not satisfy the minimum age requirement to stay alone at home in other high-income countries.^8^^,^^10^ It is warranted to develop Japanese guidelines regarding leaving children alone at home. In terms of dental caries, the risk might not increase if the frequency of being left alone at home is kept at <1 time per week. While these findings can be used to develop guidelines, other consequences of being left alone at home, such as those related to child mental health and development, must also be taken into consideration. Even though there are after-school programs for children with working parents, some children, especially in Tokyo, cannot participate in these due to overcapacity.^29^ Expanding facilities and hiring equipped staff for the after-school program would be a good solution in case of such situations as this could also help reduce the occurrence of dental caries among these children. Moreover, improving the work environment so that parents with young children can easily take a day off or leave early to take care of them would be a great relief.

The present study has several limitations. First, the causal relationship remains unclear because this is a cross-sectional study. Dental caries might have developed before the time of the surveys, and it is unknown whether the children had been left home at that time. We checked whether similar results could be observed for dental caries in permanent teeth. The temporality between exposure (ie, being left alone at home) and the outcome (ie, dental caries) would be more robust for permanent teeth, but the result showed non-significant relationships. Permanent teeth might be a less reliable outcome of the present study because they have not erupted for some children at this age. Tooth eruption can be delayed in those frequently left alone at home, considering that child neglect hinders child development.^30^ Second, the survey also included a wide range of topics that were not limited to the relationship between being left alone at home and dental caries, and the variables might not be optimal for the present study. For example, more detailed information regarding the frequency and duration of leaving children alone at home is required to assess a potential dose-response relationship. Other variables associated with dental caries, such as fluoride utilization and regular dental checkups, were not included in the analysis due to the unavailability of data. Third, the exposure variable, being left alone at home, was answered by caregivers. Lastly, the study field was one area of Tokyo. Different results might be obtained in other parts of Japan. For example, sugar consumption in rural areas is higher than that in urban areas.^31^ If we conduct a similar study in the former (ie, areas where sugar is more available to children), a greater increment in dental caries might be observed in those that are left alone at home.

In conclusion, the present study found that being left alone at home more than once a week was associated with dental caries in first graders, whereas being left alone at home less than once a week was not. Frequently leaving children alone at home may increase their dental caries. Future longitudinal studies are required to confirm these findings.

## SUPPLEMENTARY MATERIAL

eTable 1. Association between leaving alone at home and parenting practices (results from complete cases; N = 9,152)

eTable 2. Factor loadings of poor parenting behaviors (N = 9,152)

eTable 3. Frequency of missing data for each variable among the respondents included in the analysis (N = 12,029)

eTable 4. Association between frequency of leaving alone at home and dental caries of children; results from Multivariable Poisson regression analysis with multiple imputation

eTable 5. Association between frequency of leaving alone at home and dental caries of children; results from Poisson regression analysis after propensity score matching with complete cases

eTable 6. Association between frequency of leaving alone at home and oral health related behaviors of children; results from logistic regression analysis after propensity score matching with multiple imputation

eTable 7. Association between frequency of leaving alone at home and dental caries in permanent teeth of children; results from Poisson regression analysis after propensity score matching with complete cases
