# Supplementary material for: Being Left Alone at Home and Dental Caries of Children Aged 6–7 Years
Source: J Epidemiol. 2023 Jun 5;33(6):303–10. doi: 10.2188/jea.JE20210321 (PMC10165214; doi:10.2188/jea.JE20210321)
Supplement: Supplementary file 1 [file je-33-303-s001.pdf]

eTable 1. Association between leaving alone at home and parenting practices (results from complete cases; *N*=9,152)

		Being left alone at home			
	Total	Never	<1/week	≥1/week	
	N =9,152	*N* =4,903	*N* =3,386	*N*=863	*P*-value
Frequency of the child having breakfast					0.001
Eats every day (1)	8747 (95.6%)	4709 (96.0%)	3236 (95.6%)	802 (92.9%)	
Skips sometimes (2)	346 (3.8%)	166 (3.4%)	131 (3.9%)	49 (5.7%)	
Almost none (3)	54 (0.6%)	25 (0.5%)	17 (0.5%)	12 (1.4%)	
None (4)	5 (0.1%)	3 (0.1%)	2 (0.1%)	0 (0.0%)	
Child's consumption of vegetables					<0.001
Every breakfast and dinner (1)	1473 (16.1%)	854 (17.4%)	516 (15.2%)	103 (11.9%)	
Almost very breakfast and dinner (2)	2026 (22.1%)	1061 (21.6%)	786 (23.2%)	179 (20.7%)	
Every breakfast or dinner (3)	4768 (52.1%)	2502 (51.0%)	1789 (52.8%)	477 (55.3%)	
2–3 meals/week (4)	762 (8.3%)	426 (8.7%)	243 (7.2%)	93 (10.8%)	
<1 meals/week (5)	123 (1.3%)	60 (1.2%)	52 (1.5%)	11 (1.3%)	
Hours of watching TV on a weekday					<0.001
Never (1)	174 (1.9%)	112 (2.3%)	48 (1.4%)	14 (1.6%)	
<1 hour (2)	1992 (21.8%)	1205 (24.6%)	650 (19.2%)	137 (15.9%)	
1–2 hours (3)	3930 (42.9%)	2087 (42.6%)	1481 (43.7%)	362 (41.9%)	
2–3 hours (4)	2171 (23.7%)	1082 (22.1%)	856 (25.3%)	233 (27.0%)	
3–4 hours (5)	674 (7.4%)	324 (6.6%)	265 (7.8%)	85 (9.8%)	
4–5 hours (6)	164 (1.8%)	75 (1.5%)	63 (1.9%)	26 (3.0%)	
≥5 hours (7)	47 (0.5%)	18 (0.4%)	23 (0.7%)	6 (0.7%)	
Hours of computer games on a weekday					<0.001
Never (1)	3933 (43.0%)	2393 (48.8%)	1232 (36.4%)	308 (35.7%)	
<1 hour (2)	3517 (38.4%)	1794 (36.6%)	1415 (41.8%)	308 (35.7%)	
1–2 hours (3)	1407 (15.4%)	600 (12.2%)	615 (18.2%)	192 (22.2%)	
2–3 hours (4)	224 (2.4%)	89 (1.8%)	93 (2.7%)	42 (4.9%)	
3–4 hours (5)	56 (0.6%)	21 (0.4%)	22 (0.6%)	13 (1.5%)	
4–5 hours (6)	10 (0.1%)	4 (0.1%)	6 (0.2%)	0 (0.0%)	
≥5 hours (7)	5 (0.1%)	2 (0.0%)	3 (0.1%)	0 (0.0%)	
Child going to bed after 22:00					<0.001
No (1)	7865 (85.9%)	4207 (85.8%)	2973 (87.8%)	685 (79.4%)	
Yes (1)	1287 (14.1%)	696 (14.2%)	413 (12.2%)	178 (20.6%)	
Less attention to the child's schoolwork					<0.001
Almost every day (1)	7637 (83.4%)	4251 (86.7%)	2801 (82.7%)	585 (67.8%)	
3–4 times/week (2)	845 (9.2%)	376 (7.7%)	335 (9.9%)	134 (15.5%)	
1–2 times/week (3)	519 (5.7%)	216 (4.4%)	197 (5.8%)	106 (12.3%)	
1–2 times/month (4)	80 (0.9%)	29 (0.6%)	32 (0.9%)	19 (2.2%)	
Rarely (5)	71 (0.8%)	31 (0.6%)	21 (0.6%)	19 (2.2%)	
Less playing with the child through physical exercise					<0.001
Almost every day (1)	588 (6.4%)	363 (7.4%)	189 (5.6%)	36 (4.2%)	
3–4 times/week (2)	1023 (11.2%)	604 (12.3%)	355 (10.5%)	64 (7.4%)	
1–2 times/week (3)	4356 (47.6%)	2487 (50.7%)	1506 (44.5%)	363 (42.1%)	
1–2 times/month (4)	2059 (22.5%)	952 (19.4%)	882 (26.0%)	225 (26.1%)	
Rarely (5)	1126 (12.3%)	497 (10.1%)	454 (13.4%)	175 (20.3%)	
Less playing games with the child					<0.001
Almost every day (1)	397 (4.3%)	255 (5.2%)	112 (3.3%)	30 (3.5%)	
3–4 times/week (2)	952 (10.4%)	605 (12.3%)	298 (8.8%)	49 (5.7%)	
1–2 times/week (3)	3143 (34.3%)	1828 (37.3%)	1104 (32.6%)	211 (24.4%)	
1–2 times/month (4)	3200 (35.0%)	1545 (31.5%)	1318 (38.9%)	337 (39.0%)	
Rarely (5)	1460 (16.0%)	670 (13.7%)	554 (16.4%)	236 (27.3%)	
Less talking with the child about school					<0.001
Almost every day (1)	7633 (83.4%)	4162 (84.9%)	2829 (83.5%)	642 (74.4%)	

3–4 times/week (2)	1085 (11.9%)	545 (11.1%)	400 (11.8%)	140 (16.2%)	
1–2 times/week (3)	355 (3.9%)	160 (3.3%)	128 (3.8%)	67 (7.8%)	
1–2 times/month (4)	39 (0.4%)	19 (0.4%)	15 (0.4%)	5 (0.6%)	
Rarely (5)	40 (0.4%)	17 (0.3%)	14 (0.4%)	9 (1.0%)	
Less talking with the child about the news					0.015
Almost every day (1)	844 (9.2%)	447 (9.1%)	307 (9.1%)	90 (10.4%)	
3–4 times/week (2)	1223 (13.4%)	623 (12.7%)	490 (14.5%)	110 (12.7%)	
1–2 times/week (3)	1970 (21.5%)	1062 (21.7%)	716 (21.1%)	192 (22.2%)	
1–2 times/month (4)	1539 (16.8%)	779 (15.9%)	610 (18.0%)	150 (17.4%)	
Rarely (5)	3576 (39.1%)	1992 (40.6%)	1263 (37.3%)	321 (37.2%)	
Less cooking with the child					0.029
Almost every day (1)	114 (1.2%)	54 (1.1%)	42 (1.2%)	18 (2.1%)	
3–4 times/week (2)	420 (4.6%)	224 (4.6%)	152 (4.5%)	44 (5.1%)	
1–2 times/week (3)	1895 (20.7%)	994 (20.3%)	705 (20.8%)	196 (22.7%)	
1–2 times/month (4)	3978 (43.5%)	2159 (44.0%)	1492 (44.1%)	327 (37.9%)	
Rarely (5)	2745 (30.0%)	1472 (30.0%)	995 (29.4%)	278 (32.2%)	
Less going out with the child					<0.001
Almost every day (1)	1350 (14.8%)	823 (16.8%)	438 (12.9%)	89 (10.3%)	
3–4 times/week (2)	2356 (25.7%)	1149 (23.4%)	1001 (29.6%)	206 (23.9%)	
1–2 times/week (3)	4833 (52.8%)	2663 (54.3%)	1679 (49.6%)	491 (56.9%)	
1–2 times/month (4)	598 (6.5%)	261 (5.3%)	262 (7.7%)	75 (8.7%)	
Rarely (5)	15 (0.2%)	7 (0.1%)	6 (0.2%)	2 (0.2%)	
Hit the child's body (buttocks, hand, head, or face)					<0.001
No (1)	8563 (93.6%)	4621 (94.2%)	3166 (93.5%)	776 (89.9%)	
Yes (2)	589 (6.4%)	282 (5.8%)	220 (6.5%)	87 (10.1%)	
Yell at the child					<0.001
No (1)	6783 (74.1%)	3725 (76.0%)	2462 (72.7%)	596 (69.1%)	
Yes (2)	2369 (25.9%)	1178 (24.0%)	924 (27.3%)	267 (30.9%)	
Beat the child					<0.001
No (1)	8638 (94.4%)	4698 (95.8%)	3169 (93.6%)	771 (89.3%)	
Yes (2)	514 (5.6%)	205 (4.2%)	217 (6.4%)	92 (10.7%)	
Shut the child outside					<0.001
No (1)	8360 (91.3%)	4570 (93.2%)	3044 (89.9%)	746 (86.4%)	
Yes (2)	792 (8.7%)	333 (6.8%)	342 (10.1%)	117 (13.6%)	
Do not feed the child					0.013
No (1)	9095 (99.4%)	4881 (99.6%)	3362 (99.3%)	852 (98.7%)	
Yes (2)	57 (0.6%)	22 (0.4%)	24 (0.7%)	11 (1.3%)	
Insult the child repeatedly					<0.001
No (1)	8882 (97.0%)	4764 (97.2%)	3301 (97.5%)	817 (94.7%)	
Yes (2)	270 (3.0%)	139 (2.8%)	85 (2.5%)	46 (5.3%)	
Have a big fight in front of the child					0.150
No (1)	8867 (96.9%)	4760 (97.1%)	3280 (96.9%)	827 (95.8%)	
Yes (2)	285 (3.1%)	143 (2.9%)	106 (3.1%)	36 (4.2%)	

eTable 2. Factor loadings of poor parenting behaviors (N=9,152)

	Exploratory factor analysis with Promax rotation [a]			Confirmatory factor analysis [b,c]		
	Factor 1	Factor 2	Factor 3	Factor 1	Factor 2	Factor 3
Lower frequency of the child eating breakfast	−0.040	0.022	**0.251**			0.238
Less vegetables consumption of the child	0.097	0.002	**0.396**			0.523
Longer hours of TV watching on a weekday	−0.069	0.045	**0.452**			0.334
Longer hours of playing computer games on a weekday	−0.041	0.001	**0.386**			0.278
The child going to bed after 22:00	0.007	−0.031	**0.267**			0.265
Less attention to the child's schoolwork	**0.309**	0.027	0.017	0.316		
Less playing with the child through physical exercise	**0.579**	−0.004	−0.030	0.432		
Less playing games with the child	**0.525**	0.024	−0.009	0.424		
Less talking with the child about school	**0.283**	0.030	0.074	0.342		
Less talking with the child about the news	**0.298**	−0.033	**0.135**	0.383		
Less cooking with the child	**0.428**	−0.019	0.045	0.394		
Less going out with the child	**0.447**	−0.066	−0.097	0.361		
Hit the child's body (buttocks, hand, head, or face)	−0.059	**0.611**	0.015		0.450	
Yell at the child	0.035	**0.567**	0.033		0.530	
Beat the child	0.016	**0.455**	−0.020		0.451	
Shut the child outside	−0.005	**0.350**	−0.007		0.372	
Do not feed the child	0.044	**0.150**	−0.063		0.160	
Insult the child repeatedly	0.036	**0.320**	−0.016		0.356	
Have a big fight in front of the child	0.020	**0.212**	0.018		0.246	

Factor 1: poor involvement
Factor 2: child abuse
Factor 3: lack of supervision on child's health behavior
a Factor loading >0.1 or <−0.1 are shown in bold.
b Model fit indicators were comparative fit index=0.932; root mean square error of approximation=0.025; standardized root mean square residual=0.022
c Correlation of error terms was assumed when Pearson's rho >0.2.

eTable 3. Frequency of missing data for each variable among the respondents included in the analysis (*N*=12,029)

Variable	*N* (%) of missing data
Dental caries experience	0 (0.0%)
Leaving alone at home	0 (0.0%)
Survey year	0 (0.0%)
Age	0 (0.0%)
Sex	0 (0.0%)
Marital status of parents	329 (2.7%)
Mother's age	291 (2.4%)
Mother’s educational attainment	274 (2.3%)
Mother’s employment status	216 (1.8%)
Annual household income	678 (5.6%)
Survey respondent	151 (1.3%)
Having older siblings	0 (0.0%)
Having younger siblings	0 (0.0%)
Living with grandparents	0 (0.0%)
Caregiver’s K6 score	190 (1.6%)
Child’s snack eating habit	408 (3.4%)
Child’s frequency of drinking juice	394 (3.3%)
Frequency of the child having breakfast	15 (0.1%)
Child's consumption of vegetables	398 (3.3%)
Hours of watching TV on a weekday	23 (0.2%)
Hours of computer games on a weekday	155 (1.3%)
Child going to bed after 22:00	797 (6.6%)
Less attention to the child's schoolwork	69 (0.6%)
Less playing with the child through physical exercise	77 (0.6%)
Less playing games with the child	110 (0.9%)
Less talking with the child about school	109 (0.9%)
Less talking with the child about the news	128 (1.1%)
Less cooking with the child	125 (1.0%)
Less going out with the child	84 (0.7%)
Hit the child's body (buttocks, hand, head, or face)	114 (0.9%)
Yell at the child	106 (0.9%)
Beat the child	113 (0.9%)
Shut the child outside	116 (1.0%)
Do not feed the child	111 (0.9%)
Insult the child repeatedly	127 (1.1%)
Have a big fight in front of the child	142 (1.2%)

eTable 4. Association between frequency of leaving alone at home and dental caries of children; results from Multivariable Poisson regression analysis with multiple imputation

	Model 1		Model 2	
	MR (95% CI)	*P*-value	MR (95% CI)	*P*-value
Never (ref.) vs. <1/w	1.01 (0.98–1.05)	0.471	1.02 (0.98–1.05)	0.307
Never (ref.) vs. ≥1/w	1.12 (1.06–1.18)	<0.001	1.08 (1.03–1.14)	0.003
<1/w (ref.) vs. ≥1/w	1.11 (1.05–1.16)	<0.001	1.06 (1.01–1.11)	0.023

CI, confidence interval; MR, means ratio.
Model 1: adjusted for survey year, child's age, sex, marital status of parents, mother's age, mother's educational attainment, annual household income, survey respondent, having older siblings, having younger siblings, living with grandparents, caregiver's K6 score, and parenting practices
Model 2: model 1 + snack eating habit and frequency of drinking juice

eTable 5. Association between frequency of leaving alone at home and dental caries of children; results from Poisson regression analysis after propensity score matching with complete cases

	Model 1		Model 2	
	MR (95% CI)	*P*-value	MR (95% CI)	*P*-value
Never (ref.) vs. <1/w	1.05 (1.00–1.10)	0.060	1.06 (1.01–1.12)	0.010
Never (ref.) vs. ≥1/w	1.16 (1.07–1.25)	<0.001	1.14 (1.05–1.24)	<0.001
<1/w (ref.) vs. ≥1/w	1.13 (1.05–1.23)	<0.001	1.08 (1.00–1.17)	<0.001

CI, confidence interval; MR, means ratio.
Model 1: crude estimates after propensity score matching
Model 2: model 1 + snack eating habit and frequency of drinking juice

eTable 6. Association between frequency of leaving alone at home and oral health related behaviors of children; results from logistic regression analysis after propensity score matching with multiple imputation

	Snack: help oneself (1) vs. never/eat on time(0)		Juice: drink everyday (1) vs. less than everyday (0)	
	OR (95% CI)	*P*-value	OR (95% CI)	*P*-value
Never (ref.) vs. <1/w	0.92 (0.82–1.04)	0.173	1.00 (0.88–1.14)	0.978
Never (ref.) vs. ≥1/w	1.43 (1.17–1.75)	<0.001	1.12 (0.91–1.39)	0.295
<1/w (ref.) vs. ≥1/w	1.46 (1.21–1.76)	<0.001	1.16 (0.92–1.45)	0.207

CI, confidence interval; OR, odds ratio.
Model 1: crude estimates after propensity score matching
Model 2: model 1 + snack eating habit and frequency of drinking juice

eTable 7. Association between frequency of leaving alone at home and dental caries in permanent teeth of children; results from Poisson regression analysis after propensity score matching with complete cases

	Model 1		Model 2	
	MR (95% CI)	*P*-value	MR (95% CI)	*P*-value
Never (ref.) vs. <1/w	0.90 (0.66–1.23)	0.525	0.90 (0.65–1.24)	0.514
Never (ref.) vs. ≥1/w	0.93 (0.54–1.58)	0.786	1.31 (0.67–2.55)	0.427
<1/w (ref.) vs. ≥1/w	0.93 (0.55–1.57)	0.789	0.88 (0.49–1.56)	0.652

CI, confidence interval; MR, means ratio.
Model 1: crude estimates after propensity score matching
Model 2: model 1 + snack eating habit and frequency of drinking juice
